# Posterior migration of Ahmed glaucoma valve tube in a patient with Reiger anomaly: a case report

**DOI:** 10.1186/1471-2415-10-23

**Published:** 2010-09-17

**Authors:** Vishnu S Gupta, Harinder S Sethi, Malvika Gupta, Anuj Mehta, Shivram Singh, Pankaj Yadav, KPS Malik

**Affiliations:** 1 Room No.430, Eye OPD, VMMC and Safdarjung Hospital, New Delhi - 110029 India

## Abstract

**Background:**

To describe, a yet non-documented complication of GDI surgery (glaucoma drainage incision surgery) - anterior to posterior segment migration of Ahmed Glaucoma Valve (AGV) tube.

**Case Presentation:**

We report a young 9 year old boy, diagnosed with refractory glaucoma with Reiger anomaly. History included of poor vision in both eyes, left more than right with glare since childhood. He underwent GDI surgery with AGV implantation following which he developed posterior migration of AGV tube. The detailed ocular history, ophthalmic findings, clinical course, surgical management and development of the posterior tube migration is discussed.

**Conclusion:**

Posterior Migration of AGV tube has yet not been described. Also there is a role of expectant management of the complication in this case as evidenced by the benign course of events.

## Background

Rieger's anomaly [1] is characterized by a dysgenesis of the anterior ocular segment with peripheral iris strands, an abnormally prominent Schwalbe's line, and a stromal atrophy of the iris. Aniridia most commonly presents with decreased vision, photophobia, nystagmus, and strabismus. Because glaucoma develops later in childhood, enlargement of the cornea is not part of the presentation. Its frequent association with refractory secondary glaucoma has been noted in more than 50% cases. This warrants an early surgical intervention, but with an uncertain prognosis. Ahmed glaucoma valve [2,3] is an established modality of management in such cases with a variable success rate of 44-90%[4-7]. Multiple early and late postoperative complications [8,9] have been reported but an anterior to posterior segment migration is yet unrecorded. We report here a case of this kind with exceptional emphasis on the role of expectant management of the same.

## Case Presentation

A 9 year old child presented to the out patient services of our eye department with chief complaints of gradual, progressive loss of vision in both eyes, left more than right, over a period of two years.The patient also had primary complaint of intolerance to bright light, particularly in the right eye. His school teacher also complained of the child not taking adequate interest in class work with special obstinacy to outdoor sports. There is associated history of off and on redness in both the eyes, episodes being associated with ocular pain. The episodes often waned with or without topical medication acquired from local chemists, with pain free periods dominating the natural course.

On eliciting a detailed history, there was fleeting evidence of glare since childhood, with the child often closing his eyes in response to bright light, especially at night. There is no history of ocular irritation, itching, foreign body sensation, ocular deviation, eyelid droop, double vision or development of opacity in the cornea. There was no evidence of incidental trauma, febrile illness, dehydration, malnutrition, prolonged oral drug intake or any other cardio-renal anomaly or illness.

Child was the youngest of three siblings from a non consanguineous marriage, with a full term delivery at a local hospital, the pre or post natal course being uneventful. No similar complaints or features were noted in any of the siblings.

**Figure 1:**
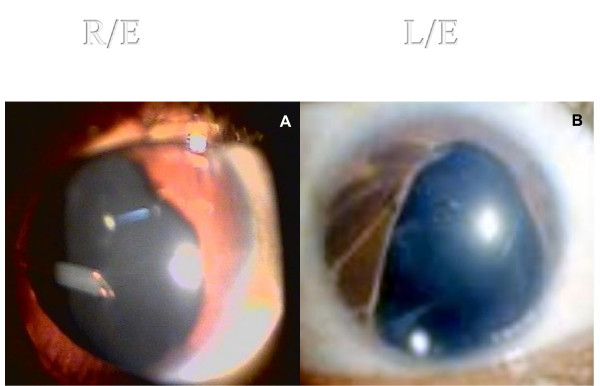
**AGV tube in situ 3 months post-operative without irido-lenticular touch**.

Local examination of the face was unremarkable. Orbits were symmetrical and of normal size and shape. Snellen's testing revealed acuity of 5/60 in the right eye whereas the child denied perception of light in the left eye. Retinoscopy was performed under cycloplegia at a distance of 1 meter, which gave an acceptance of 6/18p vision with -1.5 DS/- 2.25 DC × 120° in the right eye but left eye failed to improve. The orbit, lids, conjunctiva and cornea were essentially normal. Remarkable anomaly was noted in the iris, with stromal hypoplasia in both eyes, deforming the pupils leading to corectopia (Figure 1). Lens edge with absent zonules could be seen in superior and temporal quadrant in the right eye, in a zone of evident corectopia due to hypoplastic iris. Associated phacodonesis of crystalline lens was present, suggestive of lens subluxation. On evaluating the intraocular pressure, high values of 46 mm Hg and 64 mm Hg were seen in the right and the left eyes respectively, with Perkins applanation tonometer. Dilated fundus examination was performed. A normal size optic disc with a cup disc ratio of 0.8 was present in the right eye with well defined margins. The cup was deep with evidence of bayoneting There was mild temporal disc pallor. As for the left eye, total glaucomatous atrophy was seen.

There was no evidence of any coloboma or macular degeneration.

Gonioscopy was performed in both the eyes which showed open angles with a rudimentary trabecular meshwork with broad iris processes bridging the angle to insert on a prominent, anteriorly displaced Schwalbe's line. Areas of iris hypoplasia, with anterior insertion of iris root, were seen in both the angles.

Course of the disease and presence of useful vision in only one eye of the patient necessitated an emergent management. In view of the high intraocular pressures, the child was started on maximal anti-glaucoma therapy encompassing oral and intravenous hyperosmotic agents and topical medication with timolol and dorzolamide. The intraocular pressures fell to early thirties but continued to be raised. For such a refractory glaucoma in a young unilaterally blind child, line of management was selected to be incision surgery with aqueous shunt (AGV) implantation for the right eye and a cyclodestructive procedure for the painful blind left eye.

The surgery was uneventful with a remarkable postoperative recovery for the right eye.

The intraocular pressures fell to within 12 - 15 mm Hg with few early spikes. The left eye responded well to cyclocryotherapy with abolition of ocular pain after a time span of 6-7 weeks. The child was followed sequentially at weekly intervals for the first month after which a gradual attrition to an initial biweekly and later a monthly evaluation was maintained. The intraocular pressures were controlled and the pain free intervals prolonged. The initial squeal of congestion, heaviness and lacrimation subsided in about 3 months with a diffusely encapsulated Ahmed glaucoma valve in situ. The tube position was maintained in the anterior chamber and no irido-lenticular touch was seen (Figure 1a). No dysmotility or painful ocular movements were observed.

**Figure 2:**
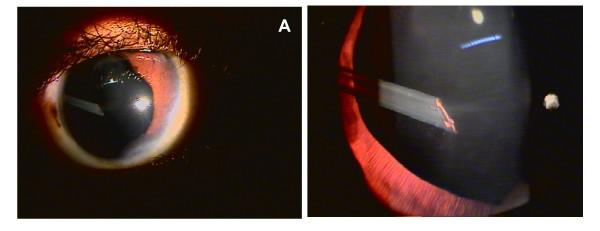
**Posterior migration of AGV tube in the vitreous cavity is evident 6 months post-operative**.

After 6 months of surgery, at his regular monthly visit, an alteration in the tube position was noted. Tube of the AGV had migrated in the posterior segment, in the anterior vitreous cavity, just posterior to the lens, abutting the posterior capsule in the superotemporal quadrant. A lens touch of the tube was evident (Figure 2a &2b). Rest of the features remained unaltered with normal intraocular pressures and no evidence of cataract.

At his current status, 12 months after surgery and 6 months after the first evidence of tube migration, the patient maintains lens transparency and normal IOP. The course in the left eye is unremarkable.

## Discussion

A syndromal mesodermal dysgenesis of the cornea and iris was described in mid 30's by Reiger [1]. In association with the prominent white line at the posterior end of the cornea, described by Axenfeld, the Reiger's anomaly consisted of iris changes such as atrophy, corectopia and hole formation. When such an anomaly was seen in association with systemic developmental defects, such as that of teeth and facial bones, it was referred to as Reiger's syndrome. General features include frequent family history, bilaterality, autosomal dominant mode of inheritance, no sex predilection and age of onset varying from birth to adulthood. High incidence of associated refractory glaucoma is noted.

The patient, who presented to us, had a similar bilateral presentation, with varying iris and corneal changes in absence of systemic developmental defects classifying him as a case of Reiger anomaly. He presented with refractory glaucoma, with an already compromised, blind left eye following total optic atrophy. The primary aim in such a young 9 year old patient is to salvage vision in the other, only seeing eye. Ahmed glaucoma valve [2,3] is an established modality of management in such cases with a variable success rate of 44- 90% [4-7].Multiple early and late postoperative complications [8] have been reported including transient hypotony (19.5%), shallow anterior chamber (14.5%), tube blockage (11.3%), hemorrhage (7.2%), encapsulated bleb (10.9%), exposure of tube (5.0%), tube malposition (4.5%), corneal decompensation (2.3%), extrusion of implant (1.4%), and rarely endophthalmitis [9], more so in pediatric age group.

Managerial decision to undertake a incision surgery with implantation of a glaucoma drainage device was undertaken in view of the above indicators. Preoperative IOP reduction was tried with maximal anti-glaucoma therapy. The incision surgery was performed in the right eye with implantation of an Ahmed Glaucoma Valve. The valve was well secured with sutures and covered by partial thickness scleral flap. The response to surgery was remarkable with good IOP control achieved within the first postoperative week. Only few high IOP spikes were noted. Visual rehabilitation was achieved early with refraction.

As for all glaucoma drainage devices, a careful observation of the tube position was noted at each postoperative visit. Tube displacement was noted at the 6th month postoperative visit. The AGV tube had migrated to the posterior segment, just posterior to the lens, abutting the posterior capsule, in the supero-temporal quadrant where iris was hypoplastic with associated corectopia. Significant touch with the posterior capsule was seen but there was no evidence of any cataractous changes. Rest of the features remained unaltered with normal intraocular pressures. Possible hypothesis of such an anterior to posterior migration may be the gravitational pull of the AGV tube in the absence of iris support (due to iris hypoplasia and corectopia) in a child of active age group. Absence of cataract formation despite evident lens touch exemplifies the superior quality and biocompatability of the polypropylene material used in AGV. Also development of cataract in such cases may not be immediate and focal lenticular changes may appear in due course.

To our knowledge, this is the first documented case report of its kind, showcasing the nature of possible posterior migration of AGV tube in a young patient with Rieger's anomaly with a clear lens despite the presence of lenticular touch.

As of now, 12 months post operative, the lens stays clear and unaltered despite the touch and a managerial decision to just observe the patient has been taken in view of one eyed status of the patient. The IOP of the patient for the right eye is within normal range. The left eye continues to have symptomatic relief post cryoablation.

## Conclusion

Posterior migration of AGV tube has yet not been described. Also there is a role of conservative non-surgical management of this complication as evidenced by the benign course of events in this case.

## Consent

Written informed consent was obtained from the father of the patient for publication of this report.

## Competing interests

The authors declare that they have no competing interests.

## Authors' contributions

VSG was the primary surgeon involved in the care of the patient and was the first to detect the posterior migration of the AGV tube at the patient's followup visit. MG performed the literature review and drafted the manuscript. HSS assisted in the final preparation and submission of the manuscript. PY helped in the investigations and management of the case. SRS assisted in AGV surgery and followup care. AM and KPSM gave the overall guidance and were involved in patient care. All authors have read and approve the final manuscript.

## Pre-publication history

The pre-publication history for this paper can be accessed here:

http://www.biomedcentral.com/1471-2415/10/23/prepub
